# Antioxidant Activity of a Sicilian Almond Skin Extract Using In Vitro and In Vivo Models

**DOI:** 10.3390/ijms241512115

**Published:** 2023-07-28

**Authors:** Alessia Arangia, Agnese Ragno, Marika Cordaro, Ramona D’Amico, Rosalba Siracusa, Roberta Fusco, Francesca Marino Merlo, Antonella Smeriglio, Daniela Impellizzeri, Salvatore Cuzzocrea, Giuseppina Mandalari, Rosanna Di Paola

**Affiliations:** 1 Department of Chemical, Biological, Pharmaceutical and Environmental Sciences, University of Messina, 98166 Messina, Italy; alessia.arangia@studenti.unime.it (A.A.);; 2 Department of Biomedical, Dental, Morphological and Functional Imaging, University of Messina, 98125 Messina, Italy; 3 Department of Pharmacological and Physiological Science, Saint Louis University School of Medicine, Saint Louis, MO 63104, USA; 4 Department of Veterinary Sciences, University of Messina, 98168 Messina, Italy

**Keywords:** paw edema, almond skin, antioxidant, inflammation, polyphenols

## Abstract

Almond skins are known for their antioxidative and anti-inflammatory properties, which are mainly due to the presence of polyphenols. The aim of the present study was to evaluate the antioxidant and anti-inflammatory effects of almond skin extract (ASE) obtained from the Sicilian cultivar “Fascionello” and to evaluate the possible mechanisms of action using an in vitro model of human monocytic U937 cells as well as an in vivo model of carrageenan (CAR)-induced paw edema. The in vitro studies demonstrated that pretreatment with ASE inhibited the formation of ROS and apoptosis. The in vivo studies showed that ASE restored the CAR-induced tissue changes; restored the activity of endogenous antioxidant enzymes, such as superoxide dismutase, catalase, and glutathione; and decreased neutrophil infiltration, lipid peroxidation, and the release of proinflammatory mediators. The anti-inflammatory and antioxidant effects of ASE could be associated with the inhibition of the pro-inflammatory nuclear NF-κB and the activation of the nuclear factor-erythroid 2-related factor 2 (Nrf2) antioxidant pathways. In conclusion, almond skin could reduce the levels of inflammation and oxidative stress and could be beneficial in the treatment of several disorders.

## 1. Introduction

The search for molecules of natural origin with anti-inflammatory and antioxidant activity is becoming increasingly popular due to the fact that natural products are safe, effective, biocompatible, and inexpensive alternatives for the treatment of inflammatory diseases [1]. Of note, there has been an increased focus on the antioxidant-rich diet, which can be a cost-effective approach that can counteract the effects of cellular aging caused by oxidative stress [1,2,3]. The consumption of functional foods, bioactive molecules, and nuts can be of great help in reducing oxidative damage; this is due to the presence of molecules with potent antioxidant activities, such as polyphenols, flavonoids, tannins, terpenoids, and anthraquinones [4,5,6,7,8]. Bioactive molecules of plant origin are currently used as alternative therapeutic agents to either prevent or treat various health problems, including inflammation and its related disorders. The regular consumption of nuts has been linked with health-promoting effects [9]. For example, almonds (*Prunus dulcis* Mill. DA Webb, also known as *Prunus amygdalus* Batsch or *Amygdalus communis* L.), are an energy-dense food containing lipids, proteins, and carbohydrates, as well as several minor bioactive compounds. They are known as a source of essential nutrients, and they are a healthy and widely researched food. The characterization of their macro- and micro-nutrients has revealed that their skin has many anti-inflammatory and antioxidant activities due to the presence of nutrients, such as fatty acids, lipids, amino acids, proteins, carbohydrates, vitamins, and minerals. The production area of the Avola almond is concentrated within the provinces of Syracuse and Ragusa; the soil and climatic characteristics of the area, as well as the seasonality and climatic changes in recent years, could be responsible for the significant variability recorded in its polyphenolic profile [10]. Almond consumption has been linked with exerting health-protective effects, and its potential prebiotic effect should also be considered [11]. In particular, the characterization of its skin has been extensively studied for the presence of anthocyanins, carotenoids, flavonoids, and other polyphenols [12,13]. The large-scale production of almonds, due to their high nutritional value as food, generates tons of waste annually. The remaining parts (the skin, hulls, etc.) have been negligibly explored. The interest in by-products has been increasing as they possess beneficial properties caused by the presence of bioactive compounds. Inadequate industrial procedures do not value by-products, such as almond skins, and they are used as cattle feed with an increased energy waste. The phytochemical profile of the almond skin suggests that it can be used as a functional food as a source of bioactive substances; as a dietary supplement; and in cosmetic formulations [14]. The use of a by-product, including almond skin, not only has an important health impact, but could also have a lower environmental impact, and help industries in reducing their waste disposal costs. The almond skin possesses a phytochemical profile that suggests that it has the potential to be a functional food that could serve as a valuable source of bioactive substances. It can be utilized as a dietary supplement and be incorporated into cosmetic formulations, and it offers various health benefits. By utilizing almond skin as a by-product, not only can industries reduce waste disposal costs, but they can also minimize their environmental impact. Moreover, almond skin is a notable source of dietary fiber and vitamins, which play a role in preventing chronic diseases and safeguarding against oxidative stress and inflammation. Numerous studies have already demonstrated the diverse biological effects of almonds and their by-products within the almond processing industry [15,16,17,18,19,20]. These effects encompass anti-inflammatory, antioxidant, antiangiogenic, antimicrobial, and antiproliferative actions. Recently, we assessed the composition and biological properties of blanched skin and blanch water, which are by-products originating from industrial blanching that were obtained from different Sicilian almond cultivars [21]. Almonds and almond skins are abundant in bioactive molecules, particularly polyphenols, flavonoids, tocopherols, tannins, fibers, minerals, amino acids, and fatty acids [22,23,24,25,26]. Notably, almond skin extract (ASE) has shown promising effects in preventing cellular aging and enhancing antioxidant activity. These benefits have been attributed to the richness of the polyphenols and flavonoids present in the almond skin [27]. Due to concerns regarding the potential side effects of the chemical compounds that are used to improve inflammatory conditions, there is a growing interest in utilizing natural alternatives, including functional and nutraceutical products [28]. Given the anti-inflammatory and antioxidant properties of the ASE, the aim of the present study was to evaluate its possible action in the acute inflammation of CAR-induced paw edema, which is a common experimental model that has been used to explore the potential anti-inflammatory effects of novel agents [29].

## 2. Results

### 2.1. Results of the Polyphenol Profile of the ASE

The phytochemical characterization of the ASE, which was obtained using reverse-phase high-performance liquid chromatography coupled with diode array detection, electrospray ionization, and mass spectrometry (RP-HPLC-DAD-ESI-MS), has been reported in Table 1. Twenty-one polyphenols belonging to several classes were identified as follows: flavanols (51.38%), flavonols (35.88%), phenolic acids (8.82%), and flavanones (3.32%). Isorhamnetin-3-*O*-glucoside was the most abundant compound, followed by catechin, epicatechin, naringenin-7-*O*-glucoside, and protocatechuic acid. The natural almond skin (NS) polyphenolic profile elucidated here differs from that previously reported by Smeriglio et al. (2016) for NS cv. Pizzuta [30].

### 2.2. In Vitro Assessment of the Cytotoxicity and Antioxidant Properties of the ASE

**Figure 1:**
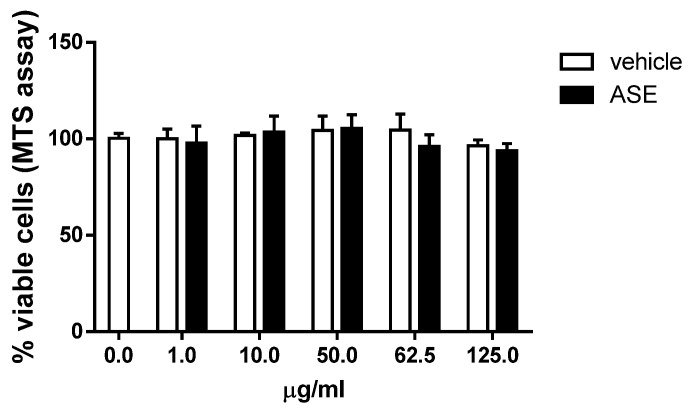
Effect of the ASE on the viability of human U937 cells. Cells were grown in 96-well microtiter plates and exposed to different concentrations of the almond skin extract (ASE) or to the corresponding quantity of the diluent DMSO (vehicle). After 24 h, cell viability was quantified with the MTS assay. Data are expressed as the mean ± SD (*n* = 3) and are represented as percentages of the 0 µg/mL-treated control (100% viability). No statistically significant differences between the groups were found.

Preliminarily, the effect of the ASE on cell viability was investigated in human monocytic U937 cells. These cells are very sensitive to chemical toxic injury and provide a useful and appropriate model for an in vitro cytotoxicity investigation. Moreover, monocytes, along with neutrophils and dendritic cells, are mostly involved in the production of cellular reactive oxygen species (ROS) during inflammation or tissue repair [31]. In order to determine a safe and effective dose of ASE in U937 cells, cell viability was assessed using the MTS assay. The U937 cells were treated with different concentrations of ASE up to 125 µg/mL or, as a control, with a medium containing the same volume of diluent as that for the treated cells (vehicle). As shown in Figure 1, the treatment with different doses of the extract did not induce evident cytotoxic effects in the range utilized in the U937 cells. In fact, even the highest dose applied did not significantly modify the percentage of viable cells compared to the untreated cells (0 µg/mL) or to the cells cultured with the diluent of the ASE (vehicle) at the same concentration.

**Figure 2:**
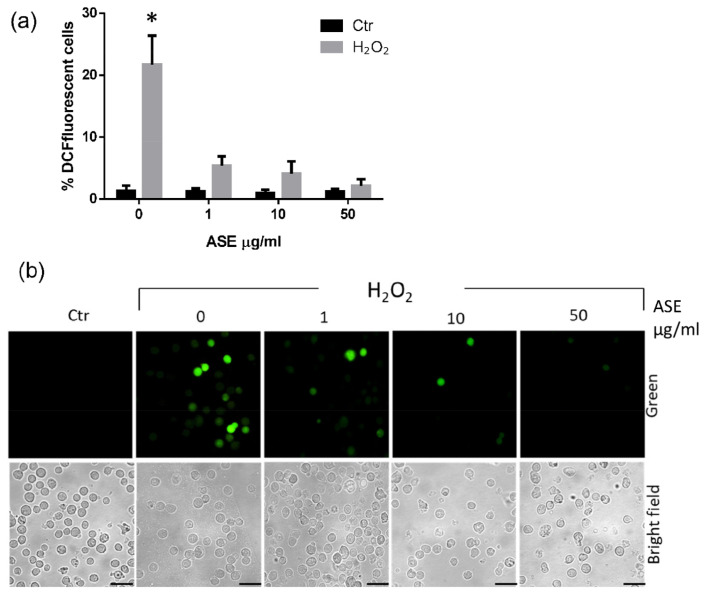
Effects of the ASE on H_2_O_2_-induced ROS generation in U937 cells. ROS generation was measured through conducting fluorescence microscopy analysis of the DCFH-DA-loaded samples. (**a**) Percentage of DCF fluorescence-positive cells. Each bar represents the mean ± SD of three independent experiments (* *p* < 0.0001. vs. all groups). (**b**) Representative images showing ROS-positive cells in the green channel and total cells in the corresponding brightfield frame. Original magnification, 400×; scale bar = 25 μm.

Based on the previous results, three concentrations of ASE, from 1 µg/mL to 50 µg/mL, respectively, were chosen to investigate the effects of this extract on cell ROS production in U937 cells. The cells were prepared at a density of 5 × 10^5^ cells/mL in a 24-well plate and cultured for 16 h at 37 °C with different concentrations of the ASE. To induce cellular oxidative stress, hydrogen peroxide (H_2_O_2_), either 5 mM or as the medium alone, was added and the cells were then incubated for a further 0.5 h. All the samples were then processed for ROS detection using a non-fluorescent agent which could be converted intracellularly into a fluorescent probe when oxidized by ROS. The results from the microscopic analysis (Figure 2) revealed that the ROS production via H_2_O_2_ stimulation was clearly inhibited in the ASE-pretreated samples, even at the lower concentrations that were tested (Figure 2a). As shown in the images of one representative analysis reported in Figure 2b, the appearance of well-detectable fluorescent cells was only easily appreciable in the H_2_O_2_ control sample, while the ASE pretreatment reduced the ROS production via H_2_O_2_ stimulation in the U937 cells.

**Figure 3:**
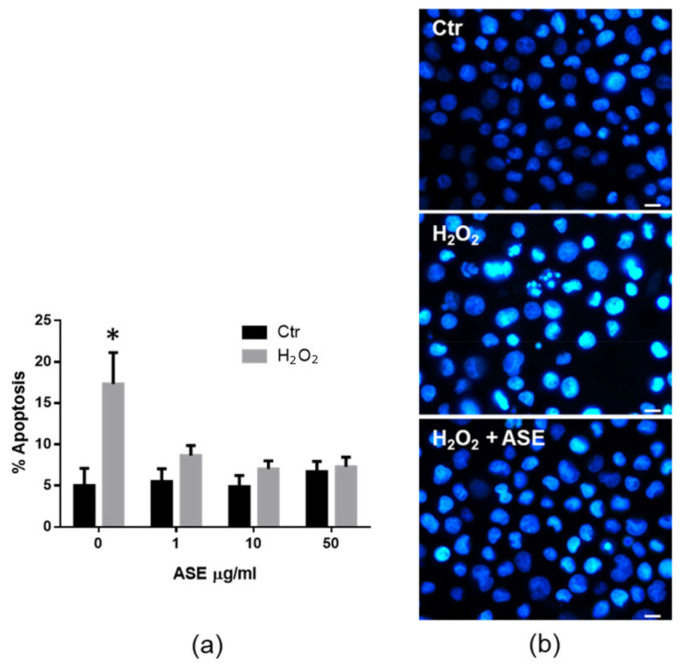
Effects of the ASE on H_2_O_2_-induced apoptosis in U937 cells. Apoptosis was evaluated as nuclear morphology changes of Hoechst-stained U937 cells assessed using fluorescence microscopy. (**a**) Percentage of cells showing features of apoptotic nuclei in each treatment group. Data are mean ± SD from three independent experiments (* *p* < 0.0001 vs. all groups). (**b**) Representative microscope images of Hoechst-stained cells after incubation with the vehicle (Ctr; upper panel), hydrogen peroxide (H_2_O_2_; middle panel), and hydrogen peroxide plus ASE (10 µg/mL) (H_2_O_2_ +ASE; lower panel). Original magnification, 630×; scale bar = 10 μm.

As an excess of ROS in the cell is known to induce apoptotic cell death [32,33], the potential role of the ASE in ROS-induced apoptosis was also examined. In these experimental panels, the U937 cells pretreated with the above concentrations of ASE for 16 h were stimulated with 0.25 mM H_2_O_2_ and analyzed after 24 h to examine for the morphological apoptotic features of the nuclei stained using Hoechst dye. The results in Figure 3a refer to the cumulative mean values of the three experiments performed and show that the ASE-pretreated cells were highly resistant to undergoing H_2_O_2_-induced apoptosis even after treatment with low concentrations of ASE. As an example, Figure 3b shows the nuclear morphology of the representative samples analyzed for apoptotic features. The nuclear morphology of the H_2_O_2_-treated cells (central panel) demonstrated an intense fluorescence resulting from increased stain absorption through condensed chromatin and visible nuclear fragmentation, which are characteristic of early and late apoptosis, respectively. As shown in the H_2_O_2_+ ASE panel, the apoptotic nuclear morphology was dramatically reduced in the U937 cells pretreated with the ASE before H_2_O_2_ treatment. Most of the controls and vehicle-treated U937 cells also exhibited a normal nuclear morphology (Ctr panel). Interestingly, we demonstrated here that ASE pretreatment protects U937 human cells from the apoptotic effects of hydrogen peroxide through corroborating its possible antioxidant activity.

### 2.3. In Vivo Studies: Effect of the ASE on CAR-Induced Inflammation and Pain

**Figure 4:**
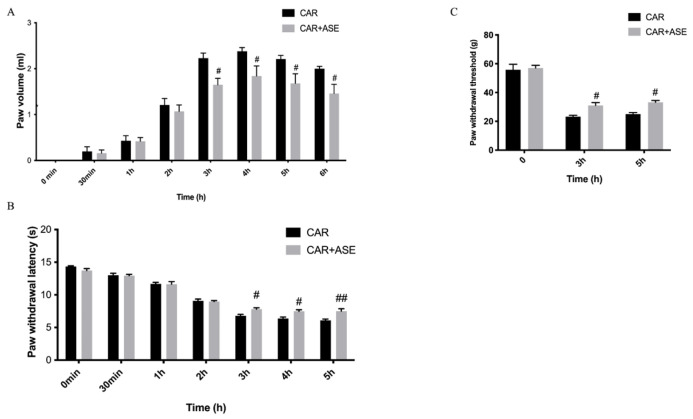
Evaluation of the effects of the ASE on CAR-induced inflammation and pain at different timepoints. Paw volume in mL (**A**), the plantar test (**B**), and the von Frey test (**C**). ASE administration showed significant improvements in the treatment of inflammation and pain. Data are expressed as means ± SEM of 6 animals for each group. # *p* < 0.05 vs. CAR; ## *p* < 0.01 vs. CAR.

One of the first visible signs of the intraplantar injection of CAR was the increase in paw volume in a time-dependent manner (Figure 4A), measured at different timepoints from 0 h (the time when the experiment started) to 6 h (the time when the experiment ended), respectively. The increase in paw volume led to the formation of pain that was assessed through the developments of thermal hyperalgesia (plantar test) and mechanical allodynia (von Frey test) (Figure 4B). In our study, we found that the injection of CAR caused an increase in thermal and mechanical hyperalgesia, while the oral treatment with ASE at the dose of 100 mg/kg given 30 min before CAR was able to significantly reduce the volume of the rat paw at 6 h post-CAR as well as decrease their pain.

### 2.4. Effects of the ASE on Histological Alteration after CAR Injection

**Figure 5:**
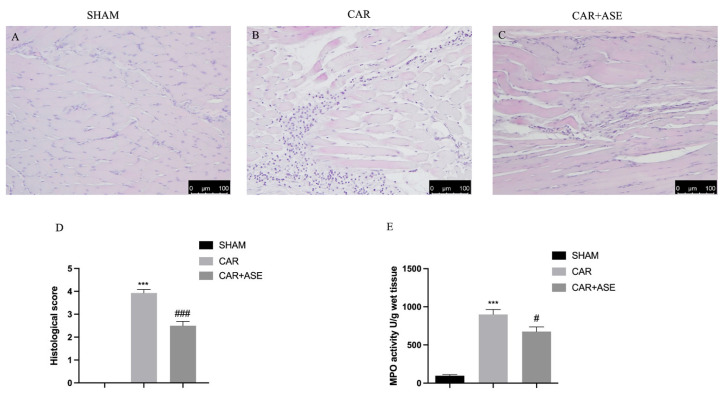
Histological evaluation of rat paw tissue and neutrophil infiltration after ASE treatment following CAR injection. H/E staining of the sham (**A**); CAR (**B**); and CAR + ASE groups (**C**). Histological score (**D**). MPO assay (**E**). Data are expressed as means ± SEM of 6 animals for each group. ### *p* < 0.001 vs. CAR; *** *p* < 0.001 vs. sham; # *p* < 0.05 vs. CAR.

At the end of the experiment, a histopathological analysis was conducted of the paw tissue with hematoxylin and eosin (H/E) examination. A microscopic study of the paw biopsies in the CAR group revealed edema formation and cellular diffusion infiltration with serious alterations in the tissue architecture (Figure 5B,D). ASE administration, at the dose of 100 mg/kg, was able to reduce the extent of histological injury in the paw tissues of the rats (Figure 5C,D), counteracting both cellular infiltration and edema formation. The sham rats showed a normal architecture of the paw tissue (Figure 5A). The presence of cellular infiltration observed with histological analysis was then also confirmed using a biochemical assay that measured the activity of myeloperoxidase (MPO), a peroxidase enzyme released by neutrophils and considered a marker of neutrophilic infiltration (Figure 5E). In our study, we found that ASE administered 30 min before a CAR injection was able to reduce the activity of MPO.

### 2.5. Effect of the ASE on Inducible NO Synthase and Cyclooxygenase-2 Enzyme Expression

**Figure 6:**
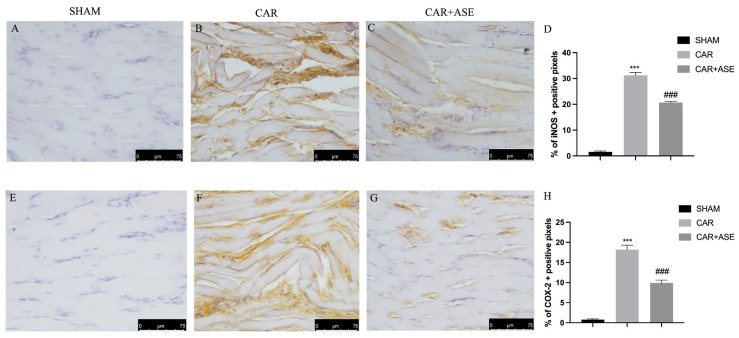
Administration of the ASE reduces the expression of iNOS and COX-2. Immunohistochemical analysis of iNOS and COX-2: sham (**A**,**E**), vehicle (**B**,**F**), and ASE treatment (**C**,**G**). The results are expressed as % of positive pixels (**D**,**H**). Figures are representative of at least three independent experiments. Values are means ± SEM of 6 animals for each group. Scale bar: 75 μm. *** *p* < 0.001 vs. sham; ### *p* < 0.001 vs. CAR.

Inducible NO synthase (iNOS) and cyclooxygenase-2 (COX-2) play primary roles in the development of inflammation; therefore, we assessed their expression through an immunohistochemistry protocol. iNOS expression was elevated in the CAR-treated animals (Figure 6B), while treatment with ASE reduced its expression and muscle fiber restoration occurred (Figure 6C); similarly, COX-2 expression was elevated in the CAR-treated animals (Figure 6F), while it was decreased in the ASE-treated animals (Figure 6G).

### 2.6. Effects of the ASE on Cytokine Production

**Figure 7:**
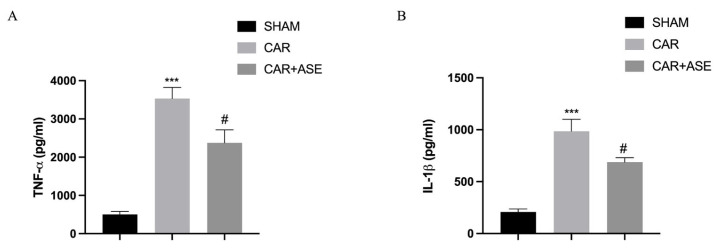
Effects of the ASE on CAR-induced cytokine production: TNF-α (**A**), and IL-1β (**B**). Values are means ± SEM of 6 animals for each group. *** *p* < 0.001 vs. sham; # *p* < 0.05 vs. CAR; # *p* < 0.05 vs. CAR; *** *p* < 0.001 vs. sham.

As well known from previous research, cytokines exert important effects during inflammatory events; for this reason, they can be used as biomarkers in indicating or monitoring inflammation and its progress [34]. In our study, we found a significant increase in serum pro-inflammatory cytokine levels of tumor necrosis factor-alpha (TNF-α) and interleukin-1β (IL-1β) in the group subjected to CAR (Figure 7A,B) compared to the sham animals. ASE administration given 30 min before CAR injection at the dose of 100 mg/kg was able to significantly decrease pro-inflammatory cytokine production.

### 2.7. Effects of the ASE on CAR-Induced Oxidative Stress

**Figure 8:**
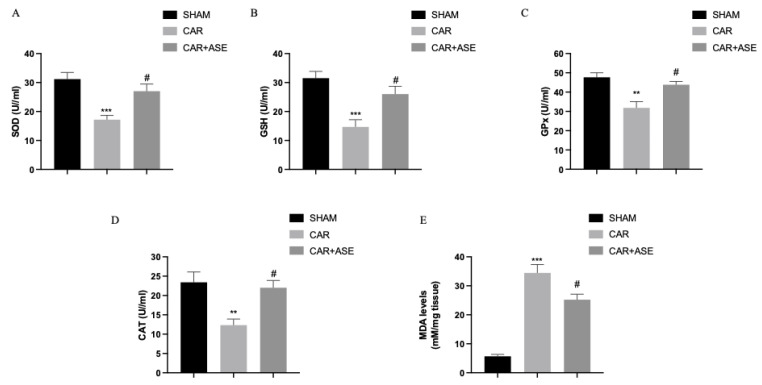
Antioxidant effects of the ASE after CAR induction: SOD (**A**), GSH (**B**), GPx (**C**), and CAT (**D**). As a consequence of lipid peroxidation, MDA was also assessed (**E**). Values are means ± SEM of 6 animals for each group. ** *p* < 0.01 vs. sham; *** *p* < 0.001 vs. sham; # *p* < 0.05 vs. CAR; # *p* < 0.05 vs. CAR.

Dietary components provide redox-active chemicals, which are useful in neutralizing the free radicals that can support the antioxidant defense system. We measured the activity of antioxidant enzymes in serum, including superoxide dismutase (SOD) (Figure 8A), glutathione (GSH) (Figure 8B), glutathione peroxidase (GPx) (Figure 8C), and catalase (CAT) (Figure 8D). We observed a decrease in the activity of SOD (Figure 8A), GSH (Figure 8B), GPx (Figure 8C), and CAT (Figure 8D) in the CAR-injected rats compared to the sham rats. The treatment with ASE was able to significantly increase the levels of SOD, GSH, GPx, and CAT. In addition, we observed increased malondialdehyde (MDA) levels as a result of oxidative stress in the CAR-injected rats treated with the vehicle (Figure 8E). The MDA levels were considerably reduced after treatment with the ASE.

### 2.8. Effect of the ASE on CAR-Induced NF-κB, Nrf2, Heme Oxygenase-1 (HO-1), and IkB-α Expression

**Figure 9:**
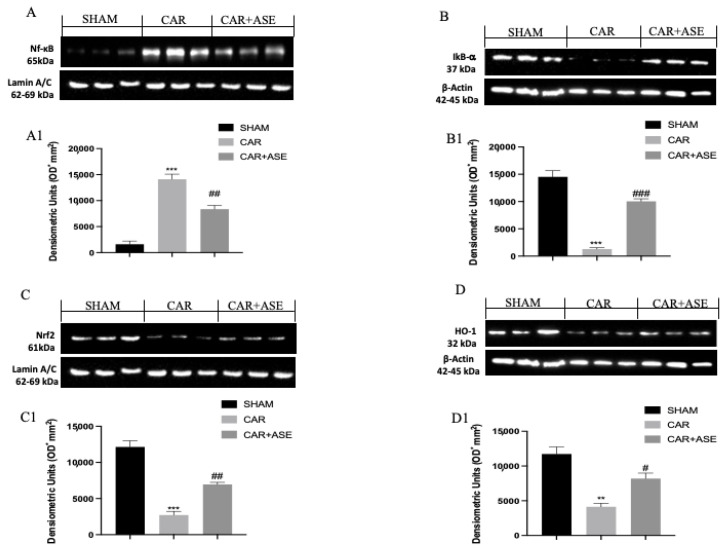
Effects of the ASE on the expression levels of NF-κB, Nrf2/HO-1, and IkB-α. Representative Western blots were performed for NF-κB, Nrf2, IkB-α, and HO-1 (**A**–**D**). We found a significant increase in NF-κB after CAR injection, compared with the sham group, along with a significant reduction in IkB-α, Nrf2, and HO-1 (**A**–**D1**). The ASE was able to reduce the expression of NF-κB and restore the expression of its inhibitor IkB-α (**A**–**B1**), Nrf2, and HO-1 (**C**–**D1**). Exposed is a blot of lysates (6 animals/group) with a densitometric analysis for all animals. ### *p* < 0.001 vs. CAR; ## *p* < 0.01 vs. CAR; # *p* < 0.05 vs. CAR; *** *p* < 0.001 vs. sham; ** *p* < 0.01 vs. sham.

To better investigate whether the ASE functions by interacting with the key signaling pathways, such as nuclear NF-κB or Nrf2/HO-1, Western blots for the NF-κB, Nrf2/HO-1, and IkB-α pathways were also performed on the paw tissues. The nuclear translocation of NF-κB was found to be increased after induction with CAR (Figure 9A), together with the reduced expression of its inhibitor IkB-α (Figure 9B; see densitometric analysis, Figure 9(B1)). Conversely, the treatment with the ASE was able to reduce the expression levels of NF-κB (Figure 9A and densitometric analysis, Figure 9(A1)) and restore the expression of IkB-α (Figure 9(B,B1)). Following CAR induction, a reduction in Nrf2 expression was observed compared with that of the sham animals (Figure 9(C,C1)). ASE increased the expression levels of Nrf2 compared with that of the CAR vehicle group (Figure 9(A,A1,C,C1)). At the same time, Western blot analysis revealed that the ASE treatment significantly increased the CAR-induced decrease in HO-1 protein expression (Figure 9(D,D1)).

## 3. Discussion

In the present study, we evaluated, for the first time, the antioxidant and anti-inflammatory effects of the ASE using in vitro and in vivo models. As shown in our previous study, the most abundant compounds in the ASE are kaempferol and its derivatives, followed by naringenin, chlorogenic acid, and vanillic acid [30]. It is well known that the production area of the Avola almond is concentrated within the provinces of Syracuse and Ragusa; the pedo-climatic features of the territory, as well as the seasonality and climatic changes over the last few years, could be responsible for the significant variability recorded in the polyphenolic profile [10]. In the present in vitro study, we demonstrated that the pretreatment with the ASE in U937 monocytic cells was able to reduce H_2_O_2_-induced ROS production and make the cells resistant to apoptosis even at low concentrations. These preliminary results were also confirmed with in vivo studies. Our in vivo work demonstrated that the oral intake of the ASE, at a dose of 100 mg/kg in an experimental model of CAR-induced paw edema, exhibits a protective action on inflammation and oxidative stress, accentuating the interest in waste products, such as the almond skin. CAR-induced inflammation causes edema and hind paw pain in animals, leading to impaired motility and problems in hind paw use [35]. Natural almond skins led to the restoration of tissue architecture and a depletion of the enzymes involved in inflammation in a model of spinal cord injury [36]. Notably, in our study, we observed that the oral administration of ASE reduced the histological damage and inflammatory cell infiltration measured through the MPO assay, restoring the tissue architecture of the paw as a result. These results were in accordance with those of Mandalari et al. [37], in which ASE was able to reduce neutrophilic infiltration into tissues. Furthermore, the induction of paw edema with CAR caused the sensitization of primary sensory neurons [38]. In this work, ASE administration was able to significantly reduce the thermal hyperalgesia and mechanical allodynia caused by the inflammatory process. The inflammatory response is caused by the activation of the NF-κB pathway, which, upon inactivation of its inhibitor IkB-α, translocates to the nuclear level, leading to the transcription of pro-inflammatory genes [39]. Consequently, the inflammatory cascade induces the production of pro-inflammatory cytokines, such as TNF-α and IL-1β, or inflammatory enzymes, such as iNOS and COX-2 [40,41,42]. Our results showed that the oral administration of ASE reduced the degradation of IkB-α and the translocation of NF-κB into the nucleus. Here, as a consequence, there was a reduction in the production of the pro-inflammatory cytokines, including TNF-α and IL-1β. These results are in agreement with those of a previous study, in which treatment with the almond skin was observed to exhibit beneficial effects in IBD by modulating the NF-κB pathway [37]. A previous study also reported that ASE administration reduced the release of pro-inflammatory cytokines [43]. Furthermore, in our experimental model, following induction with CAR, there was an inflammatory response that resulted in the formation of immune-positive cells to COX-2 and iNOS. Both the amplification of the inflammatory response and oxidative stress were aided by the expression of COX-2 and iNOS. An in vitro study on activated macrophages also reported that the protein fraction of almonds reduced the production of pro-inflammatory cytokines, such as TNF-α and IL-1β, and also reduced the expression levels of the inflammatory enzyme indicators iNOS and COX-2 [44]. Therefore, in our study, we assessed the finding that following the administration of ASE there was a reduction in the level of immune-positive cells to COX-2 and iNOS. In fact, according to the study of Lauro et al. [43], ASE leads to a significant reduction in these inflammatory enzymes. One of the most important and dangerous consequences of CAR injection is ROS-induced damage [45]. Several studies reported the antioxidant effects of almonds, suggesting that the intake of almonds may have a beneficial role in strengthening antioxidant defenses [46,47,48]. In a study using yeast models, ASE was observed to significantly reduce the presence of ROS [27]. This led to a strengthening of the antioxidant defenses under the conditions of oxidative stress. These mechanisms may be due to the presence of the polyphenols present in ASE and their antioxidant activity. Free radicals are difficult to quantify directly in vivo; hence, it is typical to quantify a variety of molecules that can interact with these free radicals, such as lipids [49]. In our study, we evaluated the levels of lipid peroxidation caused by oxidative stress with the MDA assay. MDA is a by-product and is typically used as a marker of cell membrane damage [50]. In our study, we found how the oral administration of ASE at a dose of 100 mg/kg was able to significantly reduce lipid peroxidation. In agreement with the studies of Van-Long et al. [51], we assumed that ASE enhanced antioxidant activity due to the activation of the Nrf2 pathway. Nrf2 encompasses important activities against oxidative stress as its translocation to the nuclear level causes the transcription of cytoprotective genes, such as HO-1 [52,53]. It has been observed that the almond skin can induce the activation of the phase II detoxifying/antioxidant enzymes mediated by Nrf2 and can therefore have antioxidant and hepatoprotective effects [51]. Experimental models on diabetes-related erectile dysfunction revealed that the intake of almonds increased Nrf2 activity due to their polyphenolic profiles [54]. Following CAR administration, in our work, we observed that upon inflammation there was a reduction in the Nrf2 pathway. Therefore, our study highlighted the fact that the oral administration of ASE can lead to increased Nfr2 and HO-1. SOD is an antioxidant enzyme that converts the superoxide radical or singlet oxygen to hydrogen and molecular oxygen; CAT reduces H_2_O_2_ to water and oxygen; GSH, which is highly abundant in cells, removes electrophiles and toxic metals while protecting cells; and GPx is a selenium-dependent enzyme which protects cells from oxidative damage by forming organic peroxides [55,56]. In agreement with other studies, almonds and their constituents demonstrated a considerable reducing power, enhancing the activity of antioxidant enzymes, including SOD, GSH, GPx, and CAT [57]. In our study, following CAR induction, we detected reduced SOD, GSH, GPx, and CAT levels, while ASE treatment increased endogenous antioxidant activity. According to previous studies, these observations are also due to the activation of Nrf2 by ASE, which is involved in the antioxidant system [58].

## 4. Materials and Methods

### 4.1. Almond Skin Extract

#### 4.1.1. Sample Preparation

NS was obtained from raw almonds (*Prunus dulcis* (Mill.) D.A. Webb) of the Sicilian cultivar “Fascionello” [30]. A manual stripping process, involving repeated cycles of freezing in liquid nitrogen and thawing at RT [12], was performed. The obtained NS was milled using a stainless steel blade analytical mill (IKA^®^ A11, IKA^®^-Werke GmbH and Co. KG, Staufen, Germany) with liquid nitrogen. Powdered NS (10 g) was defatted three times with n-hexane (20 mL) for 6 h under constant agitation in order to remove the lipid fraction and to obtain a more selective polyphenol extract. After filtration on Whatman filter paper no.1, the residue was mixed with methanol/0.1% HCl (*v*/*v*, 100 mL) and extracted using sonication for 15 min (Ultrasonic Cleaner USC300TH, VWR International, Radnor, PA, USA). The sample was centrifuged (5000× *g*, 10 min, 4 °C), and the extraction procedure was repeated two more times. The methanol fractions were combined and concentrated to dryness using a rotary evaporator (Büchi R-205, Büchi, Cornaredo, Italy); the residue was dissolved in MilliQ water (20 mL) and extracted four times with ethyl acetate (20 mL). The combined organic phases were dried on anhydrous sodium sulphate for 20 min and then concentrated to dryness using a rotary evaporator. The extraction yield was 1.86%.

#### 4.1.2. Qualitative and Quantitative Analysis of Polyphenols

Polyphenol characterization was carried out using RP-HPLC-DAD-ESI-MS [59]. A fully porous silica column Luna Omega 5u PS C18 100A, 150 mm × 2.1 mm (Phenomenex, Torrance, CA, USA) was used. Elution was performed with a mobile phase consisting of 0.1% formic acid (Solvent A) and methanol (Solvent B) according to the following program: 0–3 min, 0% B; 3–9 min, 3% B; 9–24 min, 12% B; 24–30 min, 20% B; 30–33 min, 20% B; 33–43 min, 30% B; 43–63 min, 50% B; 63–66 min, 50% B; 66–76 min, 60% B; 76–81 min, 60% B; and 81–86 min, 0% B, following which it was equilibrated for 4 min. The injection volume was 5 µL. The UV–Vis spectra were recorded as ranging from 190 nm to 600 nm, respectively, and acquisition was performed under different wavelengths (260 nm, 292 nm, 330 nm, and 370 nm, respectively) in order to identify all the polyphenol classes. The experimental parameters of the mass spectrometer (ion trap, model 6320, Agilent Technologies, Santa Clara, CA, USA) operating in the negative (ESI-) and positive (ESI+) ionization modes were set as follows: 3.5 kV capillary voltage; 40 psi nebulizer (N2) pressure; 350 °C drying gas temperature; 9 L/min drying gas flow; and 40 V skimmer voltage. Acquisition was carried out in full-scan mode (90–1000 *m*/*z*). Data were acquired using Agilent ChemStation software version B.01.03 and Agilent ion trap control software version 6.2. Quantification was performed through constructing calibration curves of each compound identified using HPLC-grade (purity ≥ 95%) reference standards (Extrasynthase, Geney, France).

### 4.2. Preliminary In Vitro Studies

#### 4.2.1. Cells

Human monocytic U937 cells, originally obtained from the Istituto Zooprofilattico, Brescia, Italy, were grown and cultured at 37 °C in a 5% CO_2_ incubator, as previously described [60]. RPMI medium was supplemented with 10% fetal bovine serum (FBS), 100 units/mL penicillin, 100 mg/mL streptomycin, and 2 mM L-glutamine (all from Euroclone, Milan, Italy).

#### 4.2.2. Treatments and Reagents

For the cell treatments, ASE was initially dissolved in DMSO (10 mg/mL) and then diluted in fresh medium to a final concentration of 2 mg/mL. The solution was then sterilized by filtration through a 0.2 μm filter and added to the U937 cells, split 24 h before and seeded at the density of 0.5 × 10^6^ cells/mL in either 24-well or 96-well plates. The oxidant-sensitive dye 2′,7′-dichlorofluorescin diacetate (DCFH-DA, D6883), H_2_O_2_ (H1009), and Hoechst 33342 (14533) were all purchased from Sigma-Aldrich (St. Louis, MO, USA).

#### 4.2.3. Viability Assay

Viability was assessed using a commercial MTS tetrazolium compound colorimetric kit (Cell Titer 96 Aqueous One Solution, Promega, Madison, WI, USA), according to standard procedures. The absorbance of the formazan, produced through MTS reduction in metabolic active cells, was measured at 490 nm using an iMark™ microplate absorbance reader (Bio-Rad Laboratories Inc., Hercules, CA, USA). The results were calculated according to the formula as follows: %viable cells = 100 × [Experimental value (OD490) − background average (OD490)]/Mean value of the untreated cells (OD490).

#### 4.2.4. ROS Detection

The intracellular ROS level was determined using the 2′,7′-dichlorofluorescin diacetate (DCFH-DA), as previously described [61]. Briefly, the control and treated cells were washed in PBS and labelled with 10 μM H2DCFDA for 30 min at 37 °C away from light. Following repeated washing, the cells were immediately analyzed using Leica DMR fluorescence microscopy (Leitz, Wetzlar, Germany). For quantitative evaluation of the ROS-positive cells, digital images, collected with a brightfield or green FITC filter using 40× or 63× objectives, were analyzed using ImageJ algorithm software (Version 1.53, NIH, Bethesda, MD, USA). For each frame, the background fluorescence was eliminated, and an arbitrary fixed threshold was set. The resulting green and fluorescent-positive cells were counted, and the percentage of DCF fluorescent cells relative to the total number of cells per frame, obtained in a corresponding acquired brightfield, was calculated. The data obtained from at least three separate experiments were evaluated per condition. A minimum of two frames for each experiment were analyzed.

#### 4.2.5. Apoptosis Assay

Apoptosis was assessed through microscopy analysis of the cellular (apoptotic bodies) and nuclear (chromatin condensation, nuclear fragmentation) morphology following staining with the Hoechst 33342 chromatin dye, as previously described by several of the authors of this study [62].

### 4.3. In Vivo Studies

#### 4.3.1. Animals

Male rats (Sprague-Dawley (200–230 g, Envigo, Milan, Italy)) were used throughout this study. The University of Messina Review Board for animal care (OPBA) approved the study. All the animal experiments were in agreement with the new Italian regulations (D. Lgs 2014/26) and the EU regulations (EU Directive 2010/63).

#### 4.3.2. CAR-Induced Paw Edema

The rats were given a subplantar injection of CAR (0.1 mL/rat of a 1% suspension in saline) using a 27-gauge needle into the right hind paw following anesthesia with 5.0% isoflurane in 100% O_2_, as previously described by Morris and Britti [63,64]. The animals were sacrificed with an isoflurane overdose six hours after the CAR injection. All analyses were performed with blinded experimental groups [65].

#### 4.3.3. Experimental Groups

The rats were randomly divided into the following groups of *n* = 6:

(1) CAR + vehicle (saline): rats were subjected to CAR-induced paw edema;
(2) CAR + ASE (100 mg/kg): rats were subjected to CAR-induced paw edema, and almond skin extract (100 mg/kg) was administered 30 min before CAR;
(3) The sham-operated group underwent the same surgical procedures as the CAR group, with the exception that saline or an alternative compound was administered instead of CAR.

The tested dose was chosen based on the tests previously performed in our laboratories (see preliminary results on the effect of the dose response of ASE in the Supplementary Materials Figure S1). After sacrifice, paw tissue and blood were collected for the histological and biochemical analyses.

#### 4.3.4. Assessment of CAR-Induced Paw Edema

Edema was assessed as previously described [63]. A plethysmometer (Ugo Basile, Comerio, Italy) was used to measure the volume of the paws before and after CAR injection at 6 h intervals. Edema was measured for each animal as an increase in the paw volume (mL) from the pre-injection value following CAR injection.

#### 4.3.5. Behavioral Analysis

We used the plantar and von Frey tests to gauge the analgesic effects of ASE. We used a Basile plantar test (Ugo Basile, Varese, Italy) with a latency limit of 20 s to prevent tissue damage, and we examined the hyperalgesic reaction to heat under various periods [64,66,67]. The von Frey test (BIO-EVF4, Bioseb, Vitrolles, France) was carried out in accordance with the prior descriptions [64,68,69,70]. A force transducer with a plastic tip was contained within the apparatus. When pressure was applied to the tip, the force applied was measured. The hind legs of the plantar region were touched with the tip, and an increasing upward effort was applied until the paw was freed. The force, measured in grams, at which the rats removed their paws is known as the withdrawal threshold [38].

#### 4.3.6. MPO Activity

Paw tissues were homogenized in 0.5% hexadecyltrimethyl ammonium bromide mixed in 10 mM of potassium phosphate buffer (pH 7.0) and centrifuged at 20,000× *g* at 4 °C for 30 min. An aliquot of supernatant was given time to react with a solution of 1.6 mM tetramethylbenzidine and 0.1 mM H_2_O_2_. Using a spectrophotometer, the rate of absorbance change was observed at 650 nm. MPO activity was measured in units per gram of wet tissue weight and was defined as the quantity of enzyme that could break down 1 mM of peroxide at 37 °C in 1 min [71,71,72].

#### 4.3.7. MDA Levels

MDA levels in the paw tissues were measured to evaluate the extent of lipid peroxidation, as outlined by the authors of [73]. The tissues were homogenized in a 1.15% KCl solution. A solution composed of 200 µL of 8.1% SDS, 1500 µL of 20% acetic acid (pH 3.5), 1500 µL of 0.8% thiobarbituric acid, and 700 µL of distilled water was used. The samples were then centrifuged at 3000× *g* for 10 min after being warmed for 1 h at 95 °C. At 650 nm, the absorption was determined [71,72,74,74,75,76,77].

#### 4.3.8. Evaluation of Cytokines

At the end of the experiment, to extract serum, blood was drawn using a heart puncture and centrifuged at 3000 rpm for 10 min. Prior to analysis, the serum samples were kept at a temperature of −80 °C. A colorimetric commercial ELISA kit was used to measure the levels of TNF-α and IL-1β (R&D Systems, Minneapolis, MN, USA) [78,79]. Furthermore, SOD, GSH, GPx, and CAT serum levels were assessed in accordance with the manufacturer’s recommendations (Cusabio Biotech Co., Ltd., Wuhan, Hubei, China) [80,81,82,83,84,85,86,87].

#### 4.3.9. Histological Examination of the CAR-Inflamed Hind Paw

H/E staining was prepared for histological analysis, and the treatment regimen was not known to the observers. After administering carrageenan intraplantarly, paw biopsies were collected after 6 h. Using a scalpel, the tissue from the pads of the back paws was removed. The tissue slices were dehydrated using a graduated series of ethanol solutions, embedded in Paraplast (Sherwood Medical), and fixed in Dietrick’s solution (composed of 14.25% ethanol, 1.85% formaldehyde, and 1% acetic acid) for one week at room temperature. Hematoxylin and eosin (H&E) was used to stain the sections embedded in Paraplast, which were cut into 7 μm pieces to be examined under a microscope (Leica DM7, Milan, Italy) [88]. A five-point scale was used to determine the degree of inflammation, which consisted of the following points: none, mild, mild/moderate, moderate, moderate/severe, and severe inflammation, respectively [89,90].

#### 4.3.10. Immunohistochemistry for iNOS and COX-2

The analyses of iNOS and COX-2 immunohistochemistry were performed as previously described [91,92,93]. The slices were treated with anti-iNOS and anti-COX-2 mouse monoclonal antibodies overnight (1:100 in PBS, *v*/*v*; all from Santa Cruz Biotechnology) [94,95]. The samples were washed with PBS before being exposed to secondary antibodies for incubation [91]. A biotin-conjugated goat anti-rabbit IgG and an avidin–biotin peroxidase complex were used to identify the specific labeling (Vector Laboratories, Burlingame, CA, USA) [96]. Using a Leica DM6 microscope (Leica Microsystems S.p.A., Milan, Italy), the stained sections were examined as per standard practice [97,98].

#### 4.3.11. Western Blot Analysis

The cytosolic and nuclear fractions of the paw tissue were prepared for Western blot analysis as previously described [99]. In order to standardize this procedure, the membranes were treated with anti-NF-κB (1:100) [100], anti-IkB-α (1:100), anti-Nrf2 (1:100), anti-HO-1 (1:100), β-actin (1:500), and β-laminin (1:500) (all purchased from Santa Cruz Biotechnology, Heidelberg, Germany) [101,102,103]. Using BIORAD ChemiDocTM XRS+ software (Version 6.1.0 build 7) and the enhanced chemiluminescence (ECL) detection system reagent, signals were detected, and the relative expression of the protein bands was quantified (Bio-Rad, Milan, Italy) [104]. An image of the blot signals was input into the analysis program (Image Quant TL, v2003) [92].

#### 4.3.12. Reagents

All other materials were purchased from Sigma-Aldrich Co. Stock solutions were prepared in nonpyrogenic saline (0.9% NaCl, Baxter Healthcare Ltd., Thetford, Norfolk, UK).

#### 4.3.13. Data Analysis

All values are expressed as mean ± standard error of the mean of N observations. For the in vivo experiments, N represents the number of animals. For the experiments involving histology, the photos shown are demonstrative of at least three experiments performed on different experimental days on tissue sections collected from all the animals in each group. The results were analyzed using either the two-way ANOVA when the effect of the treatment was investigated in a time-dependent mode, or with the one-way ANOVA when the means of two or more samples were analyzed. In vitro data were also assessed using the parametric one-way analysis of variance (ANOVA). All performed analyses were followed with a Bonferroni post hoc test for multiple comparisons. In all the statistical studies, GraphPad Software Prism 8 (La Jolla, CA, USA) was used. A *p*-value of less than 0.05 was considered statistically significant; # *p* < 0.05 vs. CAR; ## *p* < 0.01 vs. CAR; ** *p* < 0.01 vs. sham; *** *p* < 0.001 vs. sham.

## 5. Conclusions

Inflammation is known to be related to oxidative processes, mainly because these processes share several common pathways, such as the cross-talk between NF-κB and Nrf2/HO-1. As oxidative stress is common to several degenerative diseases, it was hypothesized that dietary antioxidants may explain a very important protective effect. Almonds are a major source of antioxidants in diets around the world. Here, we evaluated and demonstrated in vitro and in vivo that ASE is a by-product rich in polyphenols, and that it has a great potential as a nutraceutical since it has the capacity to reduce the negative effects of oxidation.

Limitations of the study: It was difficult to compare the results of an in vitro experiment performed with the whole ASE with an in vivo experiment as the oral administration route implies that the ASE undergoes digestion followed by the modification of the product.

## Figures and Tables

**Table 1:** Qualitative and quantitative characterization of polyphenols in the natural skin (NS) extract that were obtained using RP-HPLC-DAD-ESI-MS analysis. The data, which are the mean ± the standard deviation of three independent experiments in triplicate (*n* = 3), are expressed as mg/100 g of the NS dry extract (DE).

Polyphenols	RT ^a^ (min)	λ_max_ (nm)	[M − H]^−^	mg/100 g DE ^b^
Hydroxybenzoic acids				
Protocatechuic acid	7.04	258; 293	153	38.01 ± 0.55
4-Hydroxybenzoic acid	12.00	253	137	0.29 ± 0.01
Vanillic acid	16.00	262; 291	167	30.88 ± 0.42
Hydroxycinnamic acids				
Chlorogenic acid	20.50	291; 319	353	28.28 ± 0.28
trans-*p*-coumaric acid	22.80	309	163	-
Flavanones				
Eriodictyol-7-*O*-glucoside	29.71	283	449	3.90 ± 0.08
Naringenin-7-*O*-glucoside	32.43	282	433	39.36 ± 0.67
Eriodyctiol	35.66	287	287	0.01 ± 0.00
Naringenin	40.26	289	271	0.02 ± 0.00
Flavonols				
Quercetin-3-*O*-galactoside	32.36	253; 354	463	0.52 ± 0.01
Quercetin-3-*O*-rutinoside	32.41	254; 354	609	0.08 ± 0.00
Quercetin-3-*O*-glucoside	32.64	254; 354	463	2.81 ± 0.02
Kaempferol-3-*O*-rutinoside	33.96	265; 348	593	9.20 ± 0.03
Kaempferol-3-*O*-glucoside	34.34	264; 347	447	3.33 ± 0.02
Quercetin-3-*O*-rhamnoside	34.36	257; 358	447	2.04 ± 0.01
Isorhamnetin-3-*O*-glucoside	34.88	254; 353	477	377.29 ± 1.55
Quercetin	39.39	255; 370	301	0.09 ± 0.00
Kaempferol	43.66	264; 365	285	1.02 ± 0.02
Isorhamnetin	44.47	253; 368	315	0.11 ± 0.00
Flavanols				
Catechin	18.65	279	289	388.91 ± 2.21
Epicatechin	23.64	279	289	178.92 ± 1.44

^a^ RT, retention time; ^b^ DE, dry extract.

## Data Availability

The data presented in this study are available on request from the corresponding author.

## Supplementary Materials

The following supporting information can be downloaded at: https://www.mdpi.com/article/10.3390/ijms241512115/s1.
