# Supplementary material for: Antioxidant Activity of a Sicilian Almond Skin Extract Using In Vitro and In Vivo Models
Source: Int J Mol Sci. 2023 Jul 28;24(15):12115. doi: 10.3390/ijms241512115 (PMC10418603; doi:10.3390/ijms241512115)
Supplement: Supplementary file 1 [file ijms-24-12115-s001.zip › ijms-2414392-supplementary.pdf]

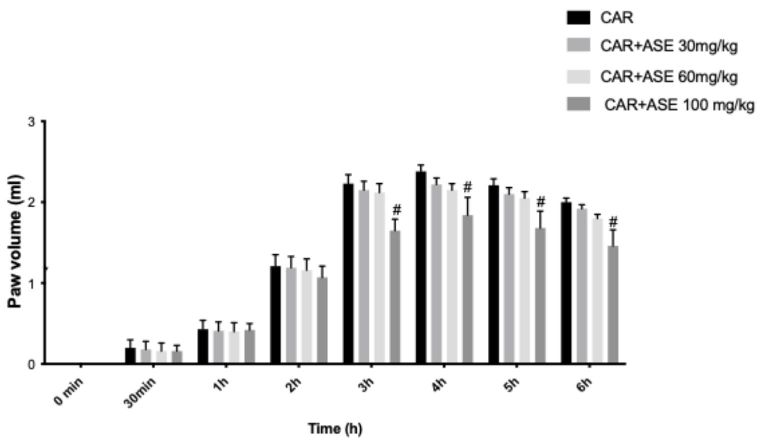

Figure S1. Preliminary data of acute effects of ASE at different doses on car-induced paw edema. The animals were treated at different doses respectively 30, 60, and 100 mg/kg. Values = means ± standard error of the mean (SEM) of six animals in each group; $^{\#}p < 0.05$ vs. Carrageenan (CAR).
